# Supplementary material for: Complexome profiling of the Chlamydomonas psb28 mutant reveals TEF5 as an early PSII assembly factor
Source: Plant Cell. 2025 Mar 17;37(6):koaf055. doi: 10.1093/plcell/koaf055 (PMC12164590; doi:10.1093/plcell/koaf055)
Supplement: koaf055_Supplementary_Data [file koaf055_supplementary_data.zip › psb28_interactive.zip!/plots/intensity/QCR9_Cre11.g468950.t1.2.html]

|  |  |
| --- | --- |
| **Identifier** | Cre11.g468950 |
| **Trivial name** | QCR9 |
| **Euclidean distance** | 35287.97 |
| **Average intensity sum (WT)** | 3480.5 || **Average intensity sum (Mut)** | 4835.9 |
| **Adjusted euclidean distance** | 7.30 |
| **MapMan annotation** | mitochondrial electron transport / ATP synthesis.cytochrome c reductase |
| **p-value** (Welch test) of intensity sums | 0.43926 |
